# A Bill Relating to Dental Practitioners in Great Britain

**Published:** 1878-03

**Authors:** 


### Editorial.

### A BILL RELATING TO DENTAL PRACTITIONERS IN GREAT BRITAIN.

In this number of the REGISTER will be found the "draft of a bill to amend the law relating to dental practitioners" in Great Briton. It will well repay a careful reading. It is interesting in several respects, but especially as showing the direction in which efforts are being made for the elevation of the profession, and the importance that attaches to a legal recognition and regulation of dental practice.

There are some important points in this bill which dentists in this country would do well to consider.

It requires that all persons who assume the title of dentist by any name shall be properly registered according to the provisions made. Any person violating this provision, is, upon conviction, subject to a fine not exceeding twenty pounds.

Without such registration no one can collect any fee or charge in any court for the performance of any dental operation, service or advice.

No one shall be registered unless he is a licentiate in dental surgery or dentistry, or who is at the passage of this act, bona fide engaged in the practice of dentistry.

A fine of from two to five pounds may be charged for registration.

Examinations are not required, but the registrar must be satisfied that the person applying for registration is entitled to be registered; but those desiring it may be examined under the supervision of the Royal College of Surgeons, of Edinburgh, or the Faculty of Physicians and Surgeons, of Glasgow, or the Royal College of Surgeons, in Ireland, and upon receiving a certificate of qualification, is entitled to registration. Every person registered under the provisions of this act shall be exempt from service on juries, inquests, etc., etc.

It is further provided that any person who falsifies in any matter relating to any register under this act, shall be deemed guilty of a misdemeanor, and is punishable by fine or imprisonment for any term not exceeding twelve months, and at the option of the general council his name be erased from the register or registers on which it appears, and any name so erased shall also be erased from the list of licentiates in dental surgery or dentistry of the college or body of which such person is a licentiate. This bill throughout is one that deserves special thought and consideration.

Mr. Jno. Tomes is the author of this bill, and the profession, not only in Great Britain but in this country also, are greatly indebted for the clear presentation of many important points

